# Safety and Efficacy of Anterior Lumbar Interbody Fusion for Discogenic Chronic Low Back Pain in a Short-stay Setting: Data From a Prospective Registry

**DOI:** 10.7759/cureus.5332

**Published:** 2019-08-07

**Authors:** Moira Vieli, Victor E Staartjes, Hubert A.J. Eversdjik, Marlies P De Wispelaere, Jan Wolter A Oosterhuis, Marc L Schröder

**Affiliations:** 1 Neurosurgery, Bergman Clinics, Amsterdam, NLD; 2 Miscellaneous, Bergman Clinics, Amsterdam, NLD; 3 General Surgery, Haaglanden Medical Center, The Hague, NLD

**Keywords:** outpatient, chronic low back pain, degenerative disc disease, clbp, alif, lumbar fusion, anterior lumbar interbody fusion, short stay, fast-track, eras

## Abstract

Introduction

As a possible treatment option for chronic lower back pain (CLBP) due to single-level degenerative disc disorder (DDD), the efficacy of anterior lumbar interbody fusion (ALIF) has been reviewed various times in the existing literature. Nevertheless, a scarcity of data exists pertaining to ALIF procedures carried out in a short-stay setting using an Enhanced Recovery after Surgery (ERAS) protocol, particularly concerning the safety.

Methods

Prospectively collected data are analyzed to study the efficacy and safety of short-stay ERAS ALIF in treatment of single-level DDD. Visual Analog Scale (VAS) in both back and leg pain along with the Oswestry Disability Index (ODI) were used to collect measure outcomes. The primary endpoint was a minimum clinically important difference (MCID) of ≥30% for the ODI at 12 months.

Results

Forty-four patients underwent surgery after failed long-term conservative treatment. MCID was achieved in 78%. Age was the only significant factor in association with MCID (p = 0.03), while gender, Modic changes, results of prognostic tests, prior surgery and smoking status had no significant influence on either MCID or change scores for any outcome measure. One complication in the form of transient new radiculopathy occurred in one patient (2.3%).

Conclusion

With overall positive outcomes in terms of both efficacy and safety, an ALIF procedure with subsequent implementation of an ERAS protocol in a short-stay setting can be an option for strictly selected patients with CLBP. Further study, however, possibly with a larger sample size, would be necessary to substantiate these findings.

## Introduction

Lumbar interbody fusion has been suggested as a treatment option for chronic low back pain (CLBP) associated with single-level degenerative disc disease (DDD) [1-4]. Through the removal of the degenerated disc as a potential pain generator and fusion of the adjacent vertebrae, the surgery aims to reduce hypermobility and microinstability, hence mitigating pain stimuli [1,5-6]. In particular, anterior lumbar interbody fusion (ALIF) has been highlighted as a promising surgical technique, since the minimum clinically important difference (MCID) in patient-reported outcomes (PROMs) can be achieved with minimal blood loss and relatively short surgical times [1-4].

The surgical treatment of CLBP due to DDD through lumbar fusion has been controversial, as it has been suggested that surgical therapy is not necessarily better than conservative therapy in the general patient population [7]. However, it has been observed that through thorough patient selection, the chances of successful treatment with interbody fusion are substantially improved, indicating that there are in fact subsets of patients that truly profit from surgery [1,8]. Especially a positive outcome of the Pantaloon Cast Test (PCT), a cast worn for a certain period of time to increase stability and therefore imitate a spinal fusion, has served as a suitable prognostic test in determining likely successful surgery in patients who have not improved through long-term conservative therapy [1,8].

Although there have been quite a few studies published on ALIF for CLBP, there is still a shortage of data regarding the safety of short-stay and outpatient ALIF procedures [9]. Enhanced recovery after surgery (ERAS) protocols [10] have also only been scarcely applied to ALIF [11-12]. Questions may be asked on outcomes and complication rates of carrying out such procedures in these settings [9]. Hence, using data from a prospective registry, we aim to evaluate the safety and efficacy of ALIF for single-level DDD in a short-stay setting, as well as to identify predictors of surgical success.

## Materials and methods

Overview

From a prospective registry, all patients who underwent elective ALIF for single-level DDD were identified. All patients were operated between March 2012 and February 2019 in a multidisciplinary team including a neurosurgeon (M.L.S.) and access surgeon (J.W.A.O.) at a specialized short-stay spine surgery clinic. Patients underwent ALIF according to the modified technique by Brau as described previously [1]. An ERAS protocol [9] was applied to improve rehabilitation after surgery, consisting of among others a strict preoperative screening and counseling for suitability of surgical treatment in this setting, thorough preoperative patient education, use of a mini-open technique and autologous cell saver transfusion, limited use of muscle relaxants during surgery, intraoperative avoidance of hypothermia, hypotension, and fluid disbalance, effective and early postoperative analgesia, early guided mobilization and elastic bracing, no restriction on activities of daily living (ADL) postoperatively, and regular, systematic audits [9,13-14] (Table 1).

**Table 1:** Elements of the institutional Enhanced Recovery after Surgery (ERAS) protocol BMI, body mass index; MI, minimally invasive; MI-PLIF, MI posterior lumbar interbody fusion; NSAIDs, non-steroidal anti-inflammatory drugs.

Timepoint	Element	Summary	Discipline
Preoperative			
1	Control of smoking and alcohol intake	Advice to cease smoking and excessive alcohol intake before surgery; Cessation of smoking 3 months before fusion surgery	Neurosurgeon
2	Strict patient screening	Strict anesthesiologic screening and patient selection to enhance perioperative patient safety by targeted optimization of comorbidities	Anesthesiologist
3	Weight loss in obese patients	Structured nutritional advice and counseling for patients with a BMI > 30	Multidisciplinary
4	Patient education	Systematic education on what to expect during recovery. Three simple principles of conduct are provided (“Three Golden Rules”)	Neurosurgeon
5	Prophylaxis against infection	Prophylaxis using a broad-spectrum antibiotic	Anesthesiologist
6	Prophylaxis against thrombosis	Prophylaxis using low molecular weight heparin	Anesthesiologist
Intraoperative			
7	Standardized anesthesia and avoidance of long-acting opioids	General anesthesia was maintained using propofol and a short-acting opioid	Anesthesiologist
8	Local analgesia	Infiltration of the surgical site with local analgesic agents, when applicable	Neurosurgeon
9	Minimally-invasive surgical techniques	By use of tubular working channels, robotic guidance, and MI or mini-open approaches, large incisions and therefore damage to muscles can be reduced	Neurosurgeon
10	Limited use of muscle relaxants	Muscle relaxants were only sparingly used to enable more efficient mobilization and recovery	Anesthesiologist
11	Prevention of fluid dysbalance and blood transfusion	Over- or underhydration was minimized, vasopressors were administered to regulate blood pressure, and autologous cell-saver transfusion was available during all procedures	Anesthesiologist
12	Prevention of hypothermia	Body temperature was controlled using warm air blankets	Anesthesiologist
Postoperative			
13	Sparing use and early removal of surgical site drains and urinary catheters	Surgical site drains were only used after mini-open decompression or MI-PLIF. Both drains and catheters were removed as early as possible	Multidisciplinary
14	Opioid-sparing analgesia	Effective analgesia was achieved using NSAIDs and paracetamol. Patient-controlled analgesia with short-acting opioids was avoided	Multidisciplinary
15	Early mobilization	Whenever feasible, patients were mobilized two hours after surgery under guidance of a physical therapist	Physical Therapist
16	Early intake of solids and fluids	Patients were encouraged to ingest oral solids and fluids at will on the day of surgery	Multidisciplinary
17	Preparation for early discharge	Integration of relatives and early organization of transport allowed patients to be discharged home early after a minimum one-night stay	Multidisciplinary
Post-discharge			
18	Minimal restriction of activities of daily living (ADL)	No restrictions in ADL were set	Neurosurgeon
19	Patient-friendly website with frequently asked questions (FAQ)	Patients are provided with a website that includes a range of FAQ as well as detailed information on the recovery process	Multidisciplinary
20	Scheduled early follow-up by phone	Two days and two weeks after surgery, patients are called to check on the status of their recovery process	Neurosurgeon
21	Low threshold for clinical follow-up visit/readmission	Patients were instructed to call in 24/7 with any uncertainties. If desired by the patient, a low threshold for a clinical follow-up visit or readmission was set	Multidisciplinary
22	Regular digital audit/follow-up	At 6 weeks, 12 months, and 24 months, questionnaires were automatically dispatched to patients digitally, allowing for effective follow-up	Multidisciplinary

Ethical considerations

This institutional registry was approved by the local institutional review board (Medical Research Ethics Committees United, Registration Number: W16.065), and this study was performed according to the Declaration of Helsinki. All individual patients in this study provided written informed consent. This study was compiled according to the Strengthening the Reporting of Observational Studies in Epidemiology (STROBE) statement [15].

Patient selection

Only patients with single-level DDD on magnetic resonance imaging (MRI) presenting with severe, intractable low back pain were considered for ALIF. The decision of a minimally invasive posterior versus anterior approach was made based upon suitable index levels, the desire to avoid the risk of impaired fertility due to the potential complication of retrograde ejaculation, and absence of spondylolisthesis. To be considered for surgical treatment, patients had to have completed ≥6 months of unsuccessful conservative treatment. Unless failed back surgery syndrome (FBSS) after at least two prior surgeries at the index level was present, patients had to undergo pantaloon cast testing (PCT) for two weeks, with a ≥50% improvement in back pain over at least 14 days to be considered for surgery [1,8,16]. Provocative discography and disco-block were not regularly used [1,17-21]. Modic type endplate changes were routinely captured, but had no influence on surgical decision-making.

Preoperative anesthesiologic screening

We applied strict selection criteria for patients considered for surgical treatment [22-23]. A very thorough anesthesiologic screening was carried out for all patients. Patients aged >80 or with a body mass index (BMI) >35, or American Society of Anesthesiologists (ASA) score >2 were not eligible for surgery [24]. These limitations are dictated by the local insurance policy. Sleep Apnea Syndrome was also a contraindication for surgery. Patients on anticoagulants were never considered for surgery in our setting. This is due to the often-associated comorbidities, and the risk of major bleeding, which represent a high surgical risk without having intensive care unit (ICU) facilities as a back-up. However, patients who were solely on acetylsalicylic acid were considered, and were allowed to continue medication perioperatively. Strict blood pressure regulation was maintained. Patients with hypertension at the preoperative screening were required to consult with their general practitioner or cardiologist to regulate blood pressure before being considered for surgery. Patients were required to cease smoking before being scheduled for fusion surgery. Osteoporosis was a contraindication for fusion procedures [25]. Patients who presented with a higher risk profile than delineated by our screening thresholds were always referred to larger, academic or community hospitals.

Perioperative management

Preoperatively, patients received cefazolin (2000 mg) as antibiotic prophylaxis, and general anesthesia was maintained using propofol and sufentanil. The use of muscle relaxants was limited to allow for faster recovery. Autologous cell-saver transfusion was available during all procedures [26]. Postoperatively, analgesia was maintained and adjusted as appropriate. All patients were mobilized early on the day of surgery under the guidance of a licensed physiotherapist and were discharged home as soon as they were able to climb stairs. Patients were provided with a light elastic brace. No restrictions on ADL were made, and patients were instructed to contact the center with any uncertainties. Patients were seen for a six-week and one-year follow-up.

Data collection

Patients completed a standardized questionnaire containing a visual analogue scale (VAS) for back pain and leg pain severity, and a validated Dutch version of the Oswestry Disability Index (ODI) as a measure of functional disability. The ODI at 12 months was defined as the primary endpoint. Patients filled in paper-based questionnaires at baseline. At six weeks and 12 months after surgery, scheduled follow-up questionnaires were obtained during the subsequent visits. All complications were systematically collected in a separate database, and reoperations were tracked.

Statistical analysis

Continuous data are given as mean ± standard deviation, and categorical data as numbers (percentages). Clinical success was defined as reaching the MCID of ≥30% improvement in ODI - the primary endpoint of this study - from baseline to the 12-month follow-up [27]. Mann-Whitney U and χ2 tests were performed to assess intergroup differences, depending on the data type. Intra-subject longitudinal data were assessed using Wilcoxon’s signed-rank test. All analyses were carried out using R version 3.5.1 (The R Foundation for Statistical Computing, Vienna, Austria). A 2-tailed p ≤ 0.05 was considered statistically significant.

## Results

Patient characteristics

In total, 44 patients were operated upon. The cohort included 15 (33%) male and 30 (67%) female patients, with the average age being 40.8 years, ranging from 19 to 62 years (Table 2).

**Table 2:** Summary of patient demographics SD, standard deviation; DDD, degenerative disc disease; PCT, pantaloon cast test; BMI, body mass index; PROM, patient-reported outcome measure; VAS, visual analogue scale; ODI, Oswestry Disability Index.

Parameter	Value (N = 44)
Age, mean ± SD [yrs.]	40.8 ± 8.8
Male gender, n (%)	15 (33)
Modic type endplate changes, n (%)	
None	8/39 (20)
1	23/39 (59)
2	8/39 (20)
Single-level DDD, n (%)	30/39 (77)
Received PCT, n (%)	33 (73)
Positive PCT, n (%)	31/33 (96)
Prior surgery, n (%)	17/40 (43)
Active smoker, n (%)	12/41 (29)
Height, mean ± SD [m]	1.77 ± 0.099
Weight, mean ± SD [kg]	76 ± 11.4
BMI, mean ± SD [kg/m^2^]	24.3 ± 2.6
History of back pain, mean ± SD [mos.]	11.2 ± 3.5
Index level, n (%)	
L3-L4	1 (2)
L4-L5	23 (51)
L5-S1	20 (42)
L4-S1	1 (2)
Baseline PROMs, mean ± SD	
VAS Back Pain	71.8 ± 19.5
VAS Leg Pain	42.9 ± 28.4
ODI	49.1 ± 14.6

Efficacy

**Table 3:** Short- and long-term outcome measures SD, standard deviation; VAS, visual analogue scale; ODI, Oswestry Disability Index; MCID, minimum clinically important difference.

Parameter	Value (N = 44)
6-week PROM scores, mean ± SD	
VAS Back pain	32.7 ± 22.4
VAS Leg pain	23.9 ± 30.5
ODI	29.5 ± 18.4
12-month PROM scores, mean ± SD	
VAS Back pain	31.4 ± 26.8
VAS Leg pain	17.9 ± 22.8
ODI	20.1 ± 18.2
12-month change score from baseline, mean ± SD	
VAS Back pain	42.6 ± 30.5
VAS Leg pain	20.7 ± 32.8
ODI	28.5 ± 22.7
MCID, n (%)	21/27 (78)

Twenty-seven patients (61%) had full data on baseline and 12-month PROMs. Clinical success was met in 21 patients, while it was not achieved in 6 patients, resulting in a clinical success rate of 78% (Table 3).

No differences were found regarding gender, age, presence of Modic changes, prior surgery or smoking status (Table 4).

**Table 4:** Analysis of factors potentially associated with MCID in the primary endpoint * p ≤ 0.05 MCID, minimum clinically important difference.

	MCID		
Parameter	NO (N = 6)	YES (N = 21)	P
Gender, n (%)			1.0
Male	2 (33)	7 (33)	
Female	4 (67)	14 (67)	
Age, n (%)			0.27
> 45	0 (0)	7 (33)	
≤ 45	6 (100)	14 (67)	
Modic Changes, n (%)			1.0
Yes	4 (67)	15 (71)	
No	1 (17)	4 (19)	
Prior surgery			0.32
Yes	2 (33)	9 (43)	
No	3 (50)	11 (52)	
Discography, n (%)			0.62
Positive	4 (67)	12 (57)	
Negative	0 (0)	3 (14)	
Smoking Status, n (%)			1.0
Smoker	2 (33)	6 (29)	
Non-Smoker	3 (50)	14 (63)	

:Table 5 lists all factors that were tested in association with PROM change scores. Out of all factors, only the association of age with ODI change score was proven to be statistically significant (p = 0.03), showing greater change scores in patients above the age of 45. Apart from that, neither gender, Modic changes, prior surgery or smoking status had an influence on the PROM change scores.

**Table 5:** Analysis of factors potentially associated with PROM change scores * p ≤ 0.05 PROM, patient-reported outcome measure; SD, standard deviation; VAS, visual analogue scale; ODI, Oswestry Disability Index; MCID, minimum clinically important difference.

Parameter	Change Score					
	ODI		VAS BP		VAS LP	
	Mean ± SD	P	Mean ± SD	P	Mean ± SD	P
Gender						
Male	27.3 ± 19.6	0.98	36.7 ± 30.0	0.52	12.9 ± 40.7	0.76
Female	28.5 ± 24.4		45.5 ± 32.3		23.7 ± 30.1	
Age						
> 45	43.4 ± 14.7	0.03*	27.1 ± 35.9	0.16	21.4 ± 29.1	0.74
≤ 45	22.8 ± 22.6		47.8 ± 27.5		20.4 ± 35.0	
Modic Changes						
Yes	29.4 ± 21.0	0.94	46.7 ± 30.4	0.49	23.5 ± 33.0	0.89
No	26.4 ± 21.0		38.0 ± 25.6		26.7 ± 23.1	
Prior Surgery						
Yes	30 ± 17.1	0.85	45.3 ± 36.4	0.70	20.8 ± 45.3	0.93
No	29.6 ± 24.5		37.7 ± 27.4		23.0 ± 23.1	
Discography						
Positive	30.8 ± 25.4	0.74	38.1 ± 35.0	1.00	12.3 ± 28.6	0.20
Negative	32.0 ± 2.0		43.3 ± 25.2		33.3 ± 11.5	
Smoking Status						
Smoker	29.5 ± 16.5	0.95	44.4 ± 27.7	0.78	7.1 ± 30.4	0.10
Non-Smoker	27.5 ± 23.6		47.4 ± 28.6		31.7 ± 29.5	

Safety

We observed only one complication. Postoperative radiculopathy appeared in one patient (2.3%), but the issue resolved after six weeks. Aside from this none of the complications associated with ALIF, such as retrograde ejaculation, incisional hernia, or vascular injuries among others, were observed in this study.

## Discussion

In this evaluation of a prospective registry, among 44 patients undergoing ALIF for degenerative disc disease using an ERAS protocol in a short-stay setting, efficacy, and safety was assessed. Clinical success was achieved in a considerable number of patients, yielding an overall clinical success rate of 78% at 12 months postoperatively. Complications were held to a minimum with the appearance of only one transient complication.

Previously, a series of RCTs demonstrated that - in a general patient population with CLBP - fusion surgery is no better than conservative treatment [7]. However, newer studies have shown that, while this finding certainly holds true in an unselected patient cohort, very strict patient selection can identify subsets of patients who truly profit from surgical treatment after years of unsuccessful conservative treatment [1]. Lammli et al. [4] found a significant decrease in pain and substantial functional improvements after the procedure. In addition, Kleimeyer et al. [2] found a higher efficacy in selective ALIF than in nonsurgical treatment. With the satisfactory outcomes regarding the effectiveness of the procedure that this study yielded for CLBP - a pain syndrome notoriously hard to treat - our findings coincide with the current literature and suggest that ALIF is effective in carefully selected patients who have failed long-term conservative treatment and when surgery is strictly considered a “last resort”.

A range of prognostic tools have been tested over the years. Apart from failed long-term conservative treatment, the PCT has shown to be the single most efficient prognostic test for outcome after surgery for CLBP, and has been independently verified to be so [1,8]. A positive PCT is especially predictive in patients with single-level DDD without prior surgery [1,8]. This effect may be explained in two ways: The PCT simulates a bony fusion by stabilizing the lumbar vertebrae, and the willingness to endure an uncomfortable pantaloon cast for two weeks indicating a certain “last resort” motivation. In this study, the predictive effects of a positive PCT, according to the definitions of its positivity (≥ 50% pain reduction), was not found by the obviously insufficient power of this specific analysis since 96% of surgically treated patients had a positive PCT. In contrast, discography has failed to show prognostic value in this patient population, and deleterious effects have even been demonstrated to occur after disc infiltration [1,17-20,28].

A low occurrence of complications in ALIF procedures, along with low morbidity have been reported by various studies [2-4,29-30]. Additionally, the success and fusion rate of the procedure has been reported to be relatively high by many authors [3]. Along with reports of shorter surgery times and lower blood loss, this substantiates the safety of ALIF procedures in a subset of the general DDD population. In light of the absence of a general or vascular surgeon, or intensive care unit in most short-stay settings, the multidisciplinary approach in partnership with a general or vascular surgeon cannot be underestimated.

While there generally is a consensus of the safety and efficacy of standalone ALIF with many studies showing similar outcomes, the results should nevertheless be interpreted with caution. Several authors have questioned the superiority of posterior spinal fusion in the long run [7]. Giang et al. [30] stated in a review of the current literature that while stand-alone ALIF is generally seen as effective, some key studies showed opposite results, fortifying the existing dissent on the topic.

ERAS protocols were first developed in colorectal surgery and have since been applied in a wide range of surgical specialties [10]. In spine surgery, ERAS has been shown to reduce acute care costs, length of hospital stay, and postoperative pain [9,12-14]. The rationale of ERAS lies within the fact that its elements aim to (1) improve the preoperative state of the patient, (2) reduce the burden of surgery, and (3) stimulate fast recovery to ADL. In our series, our proprietary ERAS protocol has been applied [9]. Although neither intergroup comparison to non-ERAS patients nor comparison to a historical control group was possible, our experience underlines the importance of a multidisciplinary team to streamline the recovery process. Foremost, this is essential in a short-stay setting, where reducing the burden of surgery can lead to massive patient and financial benefits over time, especially once the initial learning curve in implementation of the ERAS protocol has been completed [9].

Limitations

This study was largely limited by the small sample size and partly incomplete data, which resulted in low statistical power. In addition, although this was not the focus of this study, the finding pertaining to prognostic factors may be less powerful due to the low sample size, and the one statistically significant finding may have been arrived at by multiple testing. However, all data stem from a prospective registry. All procedures were conducted in a single-center, which may lead to further bias. The results may not be applicable to all treatment groups since the patients were already highly selected on the grounds of a positive PCT, and hence the analysis regarding PROMs only concern subsets of the general surgical population. In a similar manner, our findings might not be applicable to older adults, since the dataset did not include patients above the age of 62.

## Conclusions

In this study, clinical success was achieved in a considerable number of patients with the occurrence of only one complication that resolved after six weeks. With these favorable outcomes, it can be suggested that standalone ALIF in a short-stay setting with application of an ERAS protocol is an effective and safe treatment for CLBP associated with DDD. Thus, it may be an option for strictly selected patients who may profit from surgery after failed long-term conservative therapy. A multidisciplinary approach, encompassing access surgery and anesthesiology, is crucially important to the procedure’s safety and success in a short-stay setting.
